# Comparison of different commercial ELISAs for detection of antibodies against porcine respiratory and reproductive syndrome virus in serum

**DOI:** 10.1186/s12917-014-0300-x

**Published:** 2014-12-18

**Authors:** Tatjana Sattler, Eveline Wodak, Sandra Revilla-Fernández, Friedrich Schmoll

**Affiliations:** Large Animal Clinic for Internal Medicine, University of Leipzig, An den Tierkliniken 11, 04103 Leipzig, Germany; Institute for Veterinary Disease Control, AGES, Robert-Koch-Gasse 17, 2340 Mödling, Austria

**Keywords:** Swine, Wild boar, Sensitivity, Specificity, Agreement

## Abstract

**Background:**

In recent years, several new ELISAs for the detection of antibodies against the porcine reproductive and respiratory disease virus (PRRSV) in pig serum have been developed. To interpret the results, specificity and sensitivity data as well as agreement to a reference ELISA must be available. In this study, three commercial ELISAs (INgezim PRRS 2.0 - ELISA II, Priocheck® PRRSV Ab porcine – ELISA III and CIVTEST suis PRRS E/S PLUS - ELISA IV, detecting PRRSV type 1 antibodies) were compared to a standard ELISA (IDEXX PRRS X3 Ab Test - ELISA I). The serum of three pigs vaccinated with an attenuated PRRSV live vaccine (genotype 2) was tested prior to and several times after the vaccination. Furthermore, serum samples of 245 pigs of PRRSV positive herds, 309 pigs of monitored PRRSV negative herds, 256 fatteners of assumed PRRSV negative herds with unknown herd history and 92 wild boars were tested with all four ELISAs.

**Results:**

ELISAs II and III were able to detect seroconversion of vaccinated pigs with a similar reliability. According to kappa coefficient, the results showed an almost perfect agreement between ELISA I as reference and ELISA II and III (kappa > 0.8), and substantial agreement between ELISA I and ELISA IV (kappa = 0.71). Sensitivity of ELISA II, III and IV was 96.0%, 100% and 91.5%, respectively. The specificity of the ELISAs determined in samples of monitored PRRSV negative herds was 99.0%, 95.1% and 96.4%, respectively. In assumed negative farms that were not continually monitored, more positive samples were found with ELISA II to IV. The reference ELISA I had a specificity of 100% in this study.

**Conclusions:**

All tested ELISAs were able to detect a PRRSV positive herd. The specificity and sensitivity of the tested commercial ELISAs, however, differed. ELISA II had the highest specificity and ELISA III had the highest sensitivity in comparison to the reference ELISA. ELISA IV had a lower sensitivity and specificity than the other ELISAs.

## Background

The porcine reproductive and respiratory syndrome (PRRS), caused by the PRRS virus (PRRSV), is responsible for significant economic losses worldwide [1]. The PRRSV is a single strand RNA virus with high genetic variation. Two major subtypes of the virus have been described, the European genotype (type 1) and the North American genotype (type 2) [1,2]. Highly pathogenic strains that are a sub-lineage of the PRRSV type 2 were isolated in Asia [3,4]. An assessment of risk factors as well as the establishment of monitoring and surveillance programs are necessary to prevent losses due to PRRS [5]. In order to control the disease, one possible initiative is to regain a stable status in PRRSV positive herds, for instance by herd closure or mass vaccination [6,7]. Another option is the eradication of PRRSV in pig herds [8] or even in larger geographic regions [9,10]. On the other hand it is essential to maintain the status of PRRSV negative herds, for instance boar studs. Continuous and reliable monitoring of the PRRSV status of a pig herd is required in order to observe the success of the taken measures. Test systems with a high specificity and sensitivity are thus needed [11]. Several PCR methods have been established and are widely used for early diagnosis of an infection [12,13].

One cost effective method is the serological detection of antibodies against PRRSV by ELISA. Several ELISAs have recently been developed, most of them detecting antibodies against both PRRSV type 1 and type 2 [14-17]. Some ELISAs, however, are intended to be able to differentiate between type 1 and 2 antibodies [16]. The IDEXX PRRS X3 Ab Test (IDEXX, Westbrook, USA) with a sensitivity of 98.8% and a specificity of 99.9%, according to the manufacturer, is the most often cited test [1,6,14] and is generally reckoned to be the de facto gold standard of the ELISAs for detection of antibodies against PRRSV [14,15,17].

The objective of the study was to test three different commercial ELISAs for the detection of antibodies against PRRSV in serum and to evaluate their specificity and sensitivity in comparison to the IDEXX PRRS X3 Ab Test.

## Methods

### Serum samples and animals

A total of 923 serum samples of 905 pigs were included in the study. The pigs were divided into 5 groups. Group 1 consisted of 21 samples of three pigs from a PRRSV negative farm (category IV according to Holtkamp et al. [18]) that were vaccinated with attenuated live vaccine (Ingelvac PRRS MLV, Boehringer Ingelheim, Germany). Blood samples were taken from each pig before vaccination (day 0) and at day 5, 9, 12, 18, 21 and 26 after vaccination. Housing, animal care and experimental protocol were approved by the local ethics committee (Federal State Direction Saxony, Germany). Group 2 included 245 pigs from PRRSV positive farms: 49 from a boar stud in Austria, 104 fatteners from 18 Austrian farms (five to seven from each farm) with no vaccination against PRRSV, 80 further pigs (piglets, gilts and sows) from a Russian pig breeding farm and 12 pigs from a Southeast Asian pig breeding farm. Group 3 served as negative group for the evaluation of specificity of the ELISAs and included a total of 309 pigs from six monitored PRRSV negative boar studs from Germany and Austria and one German pig-breeding farm (all category IV according to Holtkamp et al. [18]). Group 4 consisted of 256 fatteners from 16 Austrian pig farms (16 samples out of each farm) that were tested once with the IDEXX PRRS X3 Ab Test (IDEXX, Westbrook, USA) with a negative result. The herd history of these farms is not known. Group 5 included serum samples of 92 hunted Austrian wild boars. The samples were taken after the death of the animal by collecting the residual blood from the thoracic cavity with subsequent centrifugation for 10 minutes at 2400 g. Table 1 gives an overview of the study groups. All samples of group 2 to 5 were collected in the course of surveillance programs and not taken for the purpose of this study.

**Table 1:** **Composition of groups included in the study and conducted analyses**

**Group**	**PRRSV herd status**	**n**	**Origin of samples**	**PCR done**
Group 1	Negative exposed	21	PRRSV vaccinated pigs	Yes
Group 2	Positive	49	Boars, Austria	Yes
		104	Fatteners, Austria	No
		80	Pigs, Russia	Yes, tissue
		12	Pigs, Southeast Asia	Yes
Group 3	Negative	153	Boars, Austria	No
		15	Sows, Germany	No
		141	Boars, Germany	No
Group 4	Suspected negative	256	Fatteners, Austria	No
Group 5	Unknown	92	Wild boars, Austria	No

### Detection of PRRSV-RNA by real-time RT-PCR

All 21 samples of group 1 were analysed by real-time RT-PCR for the presence of PRRSV viral RNA. Positive samples were sequenced. Within group 2, all serum samples of the boar stud and the Southeast Asian farm, as well as several tissue samples from the Russian farm, were analysed by PCR. Pigs from group 3 were from farms with continuous PRRSV monitoring by PCR and ELISA with no positive results. The 104 fatteners of group 2 and the samples of groups 3–5 were not tested with PCR in this study.

RNA extraction was performed using the Freedom EVO® 150 (Tecan, Grödig, Austria) automated platform and the Nucleospin® 96 Virus and the Nucleospin® Virus Core kits (Macherey-Nagel, GenXpress, Wiener Neudorf, Austria) for serum and tissue samples, respectively, following the instructions of the manufacturer. The samples were then analysed by a commercial real-time RT-PCR assay that allows the simultaneous detection and differentiation between PRRSV type 1 (EU) and type 2 (NA) genotypes (Life Technologies, Brunn am Gebirge, Austria) on the ABI 7500 Fast Real-Time PCR System (Life Technologies).

### Amplification and sequencing of the ORF5 gene

Representative PRRSV positive samples were further typed using the corresponding ORF5 gene modified methods [19,20]. Another protocol was applied for the real-time RT-PCR NA positive samples from Asia [3,4,21]. The corresponding ORF5 PCR bands of the expected sizes were excised from the agarose gel and recovered using the QIAquick® Gel Extraction Kit (Qiagen). Sequencing was performed using the BigDye® Terminator v3.1 Cycle Sequencing Kit (Life Technologies) on the 3130xl Genetic Analyzer (Life Technologies). An similarity-based tree was constructed for phylogenetical analysis based on 628 or 611 nucleotides for EU and NA ORF5 regions, respectively, using the UPGMA algorithm with BioNumerics software (version 5.1; Applied Maths, Sint-Martens-Latem, Belgium).

### Detection of PRRSV antibodies by ELISA

All serum and residual blood samples were analysed with four different commercially available ELISAs: ELISA I was the IDEXX PRRS X3 Ab test. Samples with sample-to-positive (S/P) ratios ≥0.4 (cut-off value) were considered positive for antibody against PRRSV. ELISA II was the INgezim PRRS 2.0 (Ingenasa, Madrid, Spain). The cut-off value for this ELISA was 0.4 like in ELISA I. ELISA III was the Priocheck® PRRSV Ab porcine (Prionics, Schlieren-Zurich, Switzerland). In this ELISA the cut-off value was at the S/P ratio 0.3. ELISAs I, II and III are able to detect antibodies against both type 1 and type 2 of the PRRSV. ELISA IV was the CIVTEST suis PRRS E/S PLUS (Laboratorios Hipra, Amer, Spain). The cut-off value of this ELISA was 0.2. ELISA IV is able to detect only antibodies against PRRSV type 1 . All ELISAs were conducted according to the manufacturer’s instructions.

### Statistical analysis

Positive and negative samples of group 2 were classified into two-by-two contingency tables. Sensitivity of the ELISAs compared to ELISA I was tested using the samples from group 1 and 2. The specificity of all ELISAs was estimated using group 3. The agreement of ELISA II, III and IV with ELISA I was determined using the samples of group 1 to 4 with the kappa coefficient (κ). Positive and negative predictive value, as well as the accuracy of the ELISAs were determined using group 1 to 4 with ELISA I as reference test.

## Results

### Molecular analysis

Before vaccination, all three pigs from group 1 were negative by real-time RT-PCR for both genotypes 1 and 2. Between day five and day 12 after vaccination, all three pigs were viral RNA positive for PRRSV type 2 RNA and remained negative for genotype 1. From day 18 on, all pigs were negative by PCR. Because it was known that the pigs were vaccinated with a PRRSV type 2 live vaccine, sequencing was not performed. In group 2, the boar samples were PRRSV type 1 subgroup 1 (EU-1) positive. Several field PRRSV strains belonging to PRRSV type 1 subgroup 2 (EU-2) were found in the samples originating from the Russian pig farm. Only highly pathogenic PRRSV type 2 strains were found in the Asian samples analysed.

### Detection of PRRSV antibodies by ELISA

**Figure 1:**
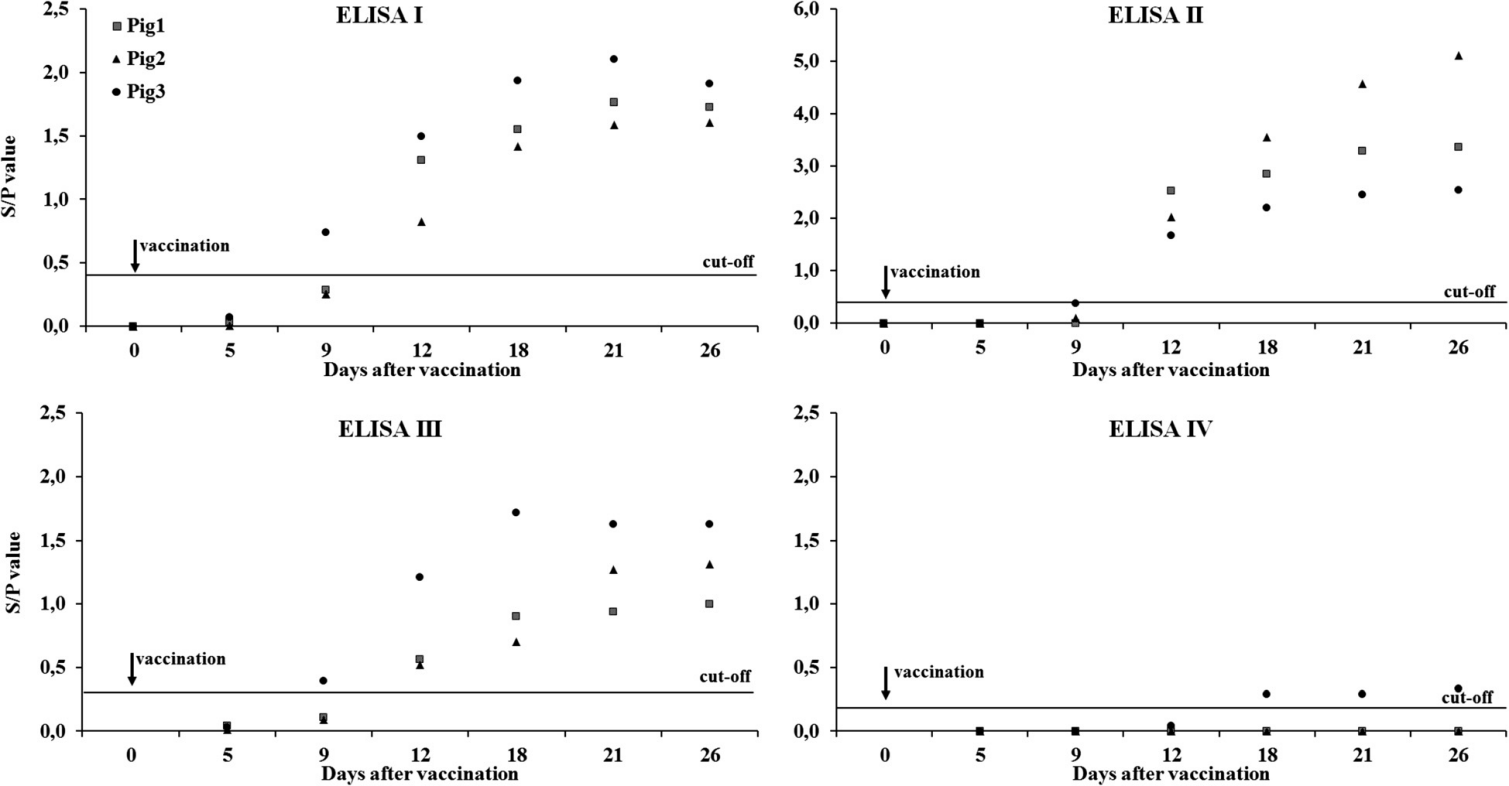
**PRRSV antibodies before and after the vaccination with a PRRSV live vaccine.** PRRSV antibodies in serum of three pigs before (day 0) and after the vaccination with a PRRSV modified live vaccine (group 1) measured with three commercial ELISAs. ELISA I - IDEXX PRRS X3 Ab test, ELISA II - INgezim PRRS 2.0, ELISA III - Priocheck® PRRSV Ab porcine, ELISA IV - CIVTEST suis PRRS E/S PLUS.

All three pigs seroconverted between day nine and day 12 after vaccination, as is seen in Figure 1. ELISA I and III detected one pig as seropositive already at day nine. In ELISA IV, which detects only PRRSV type 1 antibodies, one pig was seropositive at days 18, 21 and 26, with S/P values only slightly above the cut-off.

The S/P values of ELISA I for group 2 to 5 are displayed in Table 2. Out of the 245 samples from group 2, 189 (77%) were PRRSV antibody positive tested by ELISA I. Results of group 2 are shown in Table 3 in terms of contingency tables. Interestingly, in ELISA IV 10 samples out of 12 from the Asian farm were found positive for PRRSV type 1 antibodies, although in PCR only PRRSV type 2 RNA was detected.

**Table 2:** **Results of ELISA I (IDEXX PRRS X3 Ab test) for detection of PRRSV antibodies (mean, standard deviation (s.d.), minimum, maximum S/P values)**

**S/P value**		**Mean**	**S.D.**	**Minimum**	**Maximum**
Group 2	Positive	1.46	0.66	0.42	3.01
	Negative	0.13	0.13	0.00	0.37
Group 3		0.01	0.02	0.00	0.20
Group 4		0.06	0.07	0.00	0.39
Group 5	Negative	0.07	0.06	0.00	0.31
	Positive n = 2	-	-	0.64	2.22

**Table 3:** **Results of four commercial ELISAs for detection of PRRSV antibodies (group 2)**

		**ELISA I**		
		**Negative**	**Positive**	**Total**
ELISA II	Negative	41	7	48
	Positive	18	179	197
ELISA III	Negative	40	0	40
	Positive	19	186	205
ELISA IV	Negative	42	16	58
	Positive	17	170	187
	Total	59	186	245

Group 2: Serum samples of PRRSV positive pig farms, two-by-two contingency table. ELISA I - IDEXX PRRS X3 Ab test, ELISA II - INgezim PRRS 2.0, ELISA III - Priocheck® PRRSV Ab porcine, ELISA IV - CIVTEST suis PRRS E/S PLUS.

The results of the samples of PRRSV negative farms of group 3 are shown in Table 4. The mean S/P value of ELISA I was 0.01 in those samples can be seen in Table 2. The S/P values of the positive samples in this group, tested with ELISA II, were slightly above the cut-off (range 0.41 to 0.52). Positive samples tested with ELISA III and IV ranged from 0.30 to 1.06 and 0.21 to 0.91, respectively. In group 4, more positive samples were tested with ELISAs II, III and IV (Table 4), whereat ELISA I remained negative. S/P values of ELISA I, however, were elevated up to 0.39 in some samples, only slightly beneath the cut-off. Positive samples found in ELISAs II, III and IV were distributed among all 16 farms.

**Table 4:** **Results of four commercial ELISAs for detection of PRRSV antibodies (groups 3 and 4)**

		**ELISA I**	**ELISA II**	**ELISA III**	**ELISA IV**
Group 3	Positive	0	3	15	11
	Negative	309	306	294	298
Group 4	Positive	0	10	37	42
	Negative	256	246	219	214

Group 3: Serum of pigs from monitored PRRSV negative farms (n = 309). Group 4: Serum of pigs from farms tested negative by a single testing with ELISA (n = 256, 16 samples per farm). ELISA I - IDEXX PRRS X3 Ab test, ELISA II - INgezim PRRS 2.0, ELISA III - Priocheck® PRRSV Ab porcine, ELISA IV - CIVTEST suis PRRS E/S PLUS.

In the wild boar samples (group 5), only a few PRRSV antibody positive samples were found (Table 5). Only in some cases the samples did correspond between the ELISAs.

**Table 5:** **Results of four commercial ELISAs for detection of PRRSV antibodies (group 5)**

		**ELISA I**		
		**Negative**	**Positive**	**Total**
ELISA II	Negative	88	1	89
	Positive	2	1	3
ELISA III	Negative	87	2	89
	Positive	3	0	3
ELISA IV	Negative	74	0	74
	Positive	16	2	18
	Total	90	2	92

Group 5: Serum samples of 92 wild boars, two-by-two contingency table. ELISA I - IDEXX PRRS X3 Ab test, ELISA II - INgezim PRRS 2.0, ELISA III - Priocheck® PRRSV Ab porcine, ELISA IV - CIVTEST suis PRRS E/S PLUS.

The descriptive test parameters and agreement of the ELISAs are seen in Table 6.

**Table 6:** **Comparison of three commercial ELISAs for detection of PRRSV antibodies in pig serum**

	**ELISA II**	**ELISA III**	**ELISA IV**	**Used groups**
Sensitivity (%)	96.0	100.0	91.5	1-2 (n = 266)
Specificity (%)	99.0	95.1	96.4	3 (n = 309)
Positive predictive value (%)	86.0	73.7	66.9	1-4 (n = 828)
Negative predictive value (%)	98.7	100.0	97.4	1-4 (n = 828)
Accuracy (%)	95.3	91.4	88.2	1-4 (n = 828)
Kappa coefficient (κ)	0.88	0.83	0.71	1-4 (n = 828)

Descriptive test parameters and measures of agreement. ELISA I was used as reference test. ELISA I - IDEXX PRRS X3 Ab test, ELISA II - INgezim PRRS 2.0, ELISA III - Priocheck® PRRSV Ab porcine, ELISA IV - CIVTEST suis PRRS E/S PLUS.

## Discussion

In this study, three commercial ELISAs were compared to the IDEXX PRRS X3 Ab Test (ELISA I in this study). Two of the ELISAs (ELISA II and III) were recently developed. No published data exists concerning the specificity and sensitivity or data of agreement for these ELISAs. One recent study is available that refers to ELISA IV in comparison to ELISA I in experimentally infected pigs and 205 samples of pigs from PRRSV negative herds [16]. However, no current data for this ELISA are available for seroconversion in vaccinated pigs, pigs from naturally infected herds or wild boars.

ELISA I, II and III detected a seroconversion in all three pigs of group 1 at day 12 after vaccination with an attenuated PRRSV live vaccine. ELISA I and III, however, were able to find PRRSV antibodies in one pig as early as day 9 after vaccination. Seroconversion can usually be first detected between day 9 and 15 after vaccination or inoculation [15,16,22-25]. This was confirmed in our study with ELISA I, II and III. One pig with positive results at three occasions was found in ELISA IV which is, according to manufacturer, detecting type 1 antibodies. Since no PRRSV type 1-RNA could be detected in the pigs of group 1, this is probably a cross reaction of the ELISA. It would be useful to have more defined vaccinated or inoculated pigs to see how often this cross reaction occurs.

According to the kappa coefficient, an almost perfect agreement (κ > 0.80) was found between ELISA I on one hand and ELISA II and III on the other hand. No published reference data are available for ELISA II and III, but the newly developed ELISA II seems to be much more precise than the former INgezim PRRS Universal (Ingenasa) [26]. ELISA IV had a substantial agreement (κ = 0.71) with ELISA I that is caused by a lower specificity and sensitivity.

In our study, ELISA I as reference test had a specificity of 100%, which agrees with the manufacturer’s declaration (99.9%) and was also found in another study [16]. Other studies found only few false positive results in this ELISA [15,17]. In our study, a very high specificity (99%) was found in ELISA II as well. More false positive samples were detected in ELISA III and IV. A lower specificity of ELISA IV has also been described in other studies (93.3% and 92.5%) [16,26]. It has to be considered, however, that for determination of specificity, only group 3 was used in our study. ELISA IV tested ten out of 12 samples from Southeast Asia as PRRSV type 1 antibody positive that in PCR were found positive only for highly pathogenic PRRSV type 2. In Southeast Asia, the dominant genotype is highly pathogenic PRRSV type 2 [4]. No occurrence of PRRSV type 1 in the region of sample origin has been reported until now. Cross reactions of the antibodies between PRRSV type 1 and type 2 could be the reason for the positive results by ELISA IV. According to Stadejek et al. [2], ELISAs with coated antigens of either PRRSV type 1 or type 2 positive pigs preferentially, but not exclusively, detect antibodies of this type. The lower specificity of ELISA IV is also reflected in the lower positive predictive value, accuracy and kappa coefficient of this ELISA.

In group 4 on the other hand, some positive results were found with ELISA II and some more with ELISA III and IV, whereas ELISA I remained negative but with S/P values in some samples slightly beneath the cut-off. Since no anamnestic information is available on the herds, a PRRSV infection or previous vaccination cannot be excluded in farms of group 4 by a single testing for PRRSV antibodies. Since the sensitivity of ELISA I is, according to manufacturer, 98.9%, it may well be that the sensitivity of ELISA II and III is higher than this. On the other hand, antibody response detectable by ELISA after a PRRSV infection has a duration of at least 120 [11] to 137 days post infection [25]. Antibodies against PRRSV, detectable by ELISA, are present in a high percentage of older fatteners and sows of a positive farm [27]. In our study, the samples found positive in group 4 did not correspond between the ELISAs and concerned every farm of the group.

A slightly lower sensitivity (91.5%) of ELISA IV was found in our study, which is in agreement to the results of other studies [16,26], although the sensitivity found in these studies was even lower (66.7% and 47.1%).

Blood samples of wild boars are usually of poor quality. Therefore, the sensitivity, but more so the specificity of the ELISAs must be very high to produce reliable results. In our study, only a few wild boar samples in ELISA I, II and III were PRRSV antibody positive. This confirms the usually low seroprevalence between 0% and 3.8% in European wild boars that was shown in other studies [28-30] and confirms the high specificity and robustness of ELISAs I, II and III. ELISA IV, however, detected more wild boar samples as PRRSV antibody positive. This also agrees with the lower specificity that was found in the other groups in this study.

## Conclusions

This study shows that the newly developed ELISAs tested in this study are able to reliably detect antibodies against PRRSV in vaccinated and naturally infected pigs. The agreement to the reference ELISA I was almost perfect. ELISA II stood out due to a high specificity (99%) and sensitivity (96%). Occasional false positive results, however, need to be considered in all of the tested ELISAs, even in ELISA I that was used as reference method in this study. ELISA III has an especially high sensitivity (100%) that is probably superior to the reference ELISA I, but a lower specificity (95.1%). In ELISA IV that is on the market for several years, a substantial agreement to ELISA I was found. Both sensitivity and specificity of this ELISA were lower than in the other ELISAs.
